# T2 cytokine-driven alarmin and antiviral responses in asthma: insights into immune modulation and the role of IL-4Rα targeting

**DOI:** 10.3389/falgy.2025.1576816

**Published:** 2025-04-30

**Authors:** Jelena Pesic, Juan José Nieto-Fontarigo, Katerina Pardali, Stephen Delaney, Henric Olsson, Lena Uller

**Affiliations:** ^1^ Bioscience, Research and Early Development, Respiratory & Immunology, AstraZeneca, Gothenburg, Sweden; ^2^ Department of Experimental Medical Science, Lund University, Lund, Sweden; ^3^ Department of Biochemistry and Molecular Biology, University of Santiago de Compostela, Santiago de Compostela, Spain

**Keywords:** asthma, bronchial epithelial cells, IL-4, IL-13, IL-4Rα mAb, CCL26, IFNs, alarmins

## Abstract

**Introduction:**

Severe asthma is a heterogeneous condition characterized by distinct phenotypes and endotypes based on clinical or biological characteristics. Interleukin (IL) 4 and IL-13 are central cytokines in the type 2 (T2) immune response, crucial for T2 inflammation. Biologic therapies targeting the IL-4/IL-13 pathway, such as anti-IL-4Rα monoclonal antibodies (mAbs), have shown improvements in lung function and reductions in exacerbation rates for severe asthma. However, the precise role of early innate immune responses in mediating these therapeutic benefits remains unclear. This study investigates the acute and chronic effects of T2 cytokines on healthy and asthmatic bronchial epithelial cells (BECs), addressing the mechanisms underlying IL-4Rα mAb therapy in acute T2-driven inflammatory conditions and rhinoviral infection in asthma BECs.

**Methods:**

Human BECs from healthy and asthma donors were cultured at the air–liquid interface (ALI) and stimulated with IL-4 and IL-13, acutely or chronically, with or without IL-4Rα mAb, followed by rhinovirus (RV) infection. Cells were harvested 24 h post-infection. Expression levels of chemokines, alarmins, and antiviral mediators were quantified using RT-qPCR and multiplex ELISA.

**Results:**

CCL26 expression increased in response to IL-4 or IL-13 in healthy and asthmatic BECs, and this effect was significantly more pronounced in asthmatic BECs. IL-4Rα mAb treatment effectively inhibited CCL26 production in BECs from asthma patients. IL-4 and RV infection induced a significant increase in thymic stromal lymphopoietin (TSLP) levels in BECs from asthma compared with healthy, which was normalized by IL-4Rα mAb. No significant effects of T2 cytokines on alarmins were observed in healthy BECs. Chronic exposure to T2 cytokines following RV infection significantly decreased TSLP and IFN*λ*1 but increased IFNβ, specifically in asthmatic BECs.

**Conclusions:**

Our study on T2 cytokines' effects on BECs reveals that asthma BECs have an increased inflammatory response to IL-4 and IL-13. These responses, marked by increased CCL26 and TSLP, were effectively mitigated by IL-4Rα mAb. Importantly, this treatment maintained essential antiviral defenses, such as IFNβ, even post-rhinoviral infection. Our results suggest a novel mechanism by which IL-4Rα mAb controls exacerbations and improves lung function.

## 1 Introduction

Asthma is a complex inflammatory disease characterized by persistent airway inflammation resulting from cytokine-orchestrated signaling between different cell types (1, 2). In this context, the airway epithelium is critically involved in regulating both innate and adaptive immune responses in the lung and plays a pivotal role in the pathogenesis of asthma (3). Airway epithelial cells produce a set of epithelial-derived cytokines or alarmins [thymic stromal lymphopoietin (TSLP), Interleukin (IL) 25, and IL-33] in response to environmental triggers (1, 2). In turn, these epithelial-derived alarmins promote the recruitment and activation of various immune cells, including type 2 innate lymphoid cells (ILC2) and T helper 2 (Th2) cells, which are pivotal in type 2 (T2) inflammation (4, 5).

ILC2 and Th2 cells secrete large amounts of type 2 cytokines (T2), IL-4, IL-5, and IL-13, which are key drivers of asthma pathogenesis (2, 6). For example, IL-5 participates in the maturation, recruitment, activation, and survival of eosinophils; IL-4 is involved in IgE class-switching and Th2 cell differentiation, and IL-13 participates in airway inflammation, remodeling, and mucus hypersecretion. In addition, epithelial cells also secrete the chemokine CCL26, also known as eotaxin-3, in response to IL-4 and IL-13. CCL26 plays a significant role in asthma by attracting CCR3-expressing cells including eosinophils, basophils, and T2 lymphocytes (7). The link between CCL26 and asthma has been established in several studies, highlighting its role in disease development (8). The significant role of epithelial cells in the pathogenesis of asthma and the importance of the interaction between CCL26 and the T2 cytokines IL-4 and IL-13 and their receptors in the inflammatory response suggest that the modulation of CCL26 production may provide a novel therapeutic opportunity for reducing airway inflammation in asthma.

Environmental factors, such as allergens, pollutants, and viral infections, can further exacerbate airway inflammation in asthma (9–11). Among these, viral infections, particularly rhinoviruses (RV), are the leading cause of exacerbations (12). The underlying mechanisms involve complex interactions between inflammatory cells, cytokines, and structural cells of the airways where inflammation and mucus overproduction lead to airway hyperresponsiveness and narrowing of the airways (12). Understanding the triggers and mechanisms underlying these exacerbations is essential for effective management and prevention. Interferons (IFNs), especially type I (IFN-α/β) and type III (IFN-*λ*) IFNs, have a major role in mediating early antiviral responses (13). Impaired innate immune responses have been reported to be a possible mechanism involved in inducing asthma exacerbations in patients with asthma (14). Moreover, T2 cytokines (IL-4 and IL-13) may impair components of innate immunity, such as the production of IFN-β and IFN-*λ*, in response to rhinovirus infection (15). Altogether, substantial evidence supports the role of T2 cytokines in asthma pathogenesis and disease exacerbations. Targeting these pathways and mediators offers promising therapeutic strategies for managing asthma. For instance, blocking the action of IL-4, IL-5, and IL-13 with monoclonal antibodies has shown a reduction in airway inflammation and an improvement in asthma symptoms (16). Biologics targeting the IL-4 receptor are designed to modulate this immune response and have been successful in the treatment of severe asthma. Dupilumab is a monoclonal antibody that inhibits the signaling of IL-4 and IL-13 by binding to the shared IL-4 receptor alpha subunit (IL-4Rα). It is approved for the treatment of certain types of asthma and other inflammatory conditions (17).

This study aims to investigate the acute and chronic effects of T2 cytokines on bronchial epithelial cells (BECs) derived from healthy and/or asthma individuals. Furthermore, it examines the mechanisms underlying IL-4Rα mAb therapy in acute T2-driven inflammatory conditions and during rhinoviral infection in asthma BECs using a translational in vitro model of differentiated airway epithelial cells.

## 2 Material and methods

### 2.1 Air–liquid interface cell culture

Primary healthy or asthma human bronchial/tracheal epithelial cells (*N* = 4–6) (NHBE/D-HBE-As, Lonza, Basel, Switzerland) were expanded to Passage 2 and then seeded onto 0.4 μm Corning HTS Transwell 24-well permeable supports (Sigma-Aldrich, St. Louis, MO, USA). Cells were differentiated at the air–liquid interface (ALI) using the PneumaCult media system (STEMCELL Technologies, Vancouver, British Columbia, Canada) following the manufacturer's instructions.

### 2.2 ALI cell culture stimulation and treatment

Once fully differentiated, cells were stimulated for 24 h (acute) or 10 days (chronic) by basolateral addition of human recombinant proteins IL-4 and IL-13 (100 and 50 ng/ml, respectively; R&D Systems, Minneapolis, MN, USA) diluted in PneumaCult ALI medium. One hour after cytokine addition, cells were treated with an anti-human IL-4Rα therapeutic antibody (200 nM, Creative Biolabs, Inc., USA) or human IgG4 (200 nM, 13505-HNAH, Sino Biological, USA).

### 2.3 Human rhinovirus

Human rhinovirus serotype 1B (HRV1B, lot number H1611A) was purchased from Virapur (San Diego, CA, USA). HRV stocks were generated by infecting H1-HeLa cells, and the resulting supernatant was precipitated with polyethylene glycol (PEG), rinsed with PBS, and resuspended in DMEM, containing 1% penicillin/streptomycin fungizone, 5 mM MgCl, 20 mM HEPES-buffered saline (pH 7.2), and stored at −80°C. The infectious titer determined by the TCID50 assay was 6.3 × 10^9^ infectious units per milliliters.

### 2.4 RV infection of cell cultures

ALI cultures were incubated with IL-4/IL-13 cytokines for either 24 h or 10 days. These cultures were treated with anti-IL-4Rα mAb or IgG before infection with HRV1B at a multiplicity of infection (MOI) 0.1 or 1, administered apically. Following our established protocol, the infection process lasted for 3.5 h at 33°C. Subsequently, the cultures were washed with Dulbecco’s phosphate-buffered saline (DPBS) (without Ca and Mg) and then maintained in an incubator at 37°C until they were harvested, 24 h post-infection (Supplementary Figure S1). Cell health and viability were assessed daily by phase-contrast microscopy monitoring any effects of RV infection, such as signs of cell death or lifting. Observations focused on cellular morphology did not reveal any signs of detachment, rounding, or other alterations indicative of cellular distress or death.

### 2.5 RNA extraction and qualification of gene expression by real-time quantitative PCR

Total RNA was isolated from cell lysates using the Direct-zol 96 RNA kit (R2056, Zymo Research, Nordic BioSite) according to the manufacturer's protocol. RT-qPCR gene expression analysis was performed on a TaqMan qPCR system with standard primers from TaqMan (Thermo Fisher Scientific, Netherlands). GAPDH (HS04420632_g1) and HPRT1 (HS02800695 m1) were used as reference genes. The expression of the following target genes was analyzed: IFNβ1 (Hs01077958_s1), IFN*λ*1 (Hs00601677_g1), MDA5 (Hs00223420_m1), CCL26 (Hs00171146_m1). The 2^−ΔΔCT^ method was used to calculate relative changes in gene expression determined from real-time quantitative PCR experiments. In the present study, the data are presented as the fold change in target gene expression normalized to the geomean of control genes (GAPDH and HPRT1) and relative to the control (untreated) or IgG-treated cells.

### 2.6 Protein qualification

Cell culture supernatants were collected for protein concentration measurements. U-Plex (Meso Scale Discovery, Inc., Rockville, MD, USA) was used to determine cytokine concentrations of CCL26, TSLP, IL-33, and IL-25 (K15067M-2) following manufacturer instructions. The limit of detection was 7.3 pg/ml, 0.2 pg/ml, 0.59 pg/ml, and 1.8 pg/ml.

### 2.7 Statistical analysis

Data analysis was conducted using ordinary one-way ANOVA after confirming normality through Q–Q plots. *Post hoc* analyses were performed using Šidák's or Tukey's multiple-comparisons tests in GraphPad Prism 9.00. *P*-values less than 0.05 were considered statistically significant. Results are presented as fold changes in target gene expression, normalized to the geometric mean of housekeeping genes and relative to either the control (untreated) or IgG-treated groups. Protein levels are presented in pg/ml.

## 3 Results

### 3.1 Asthmatic BECs have pronounced inflammatory and antiviral responses following T2 stimulation and viral infection compared to healthy BECs

**Figure 1:**
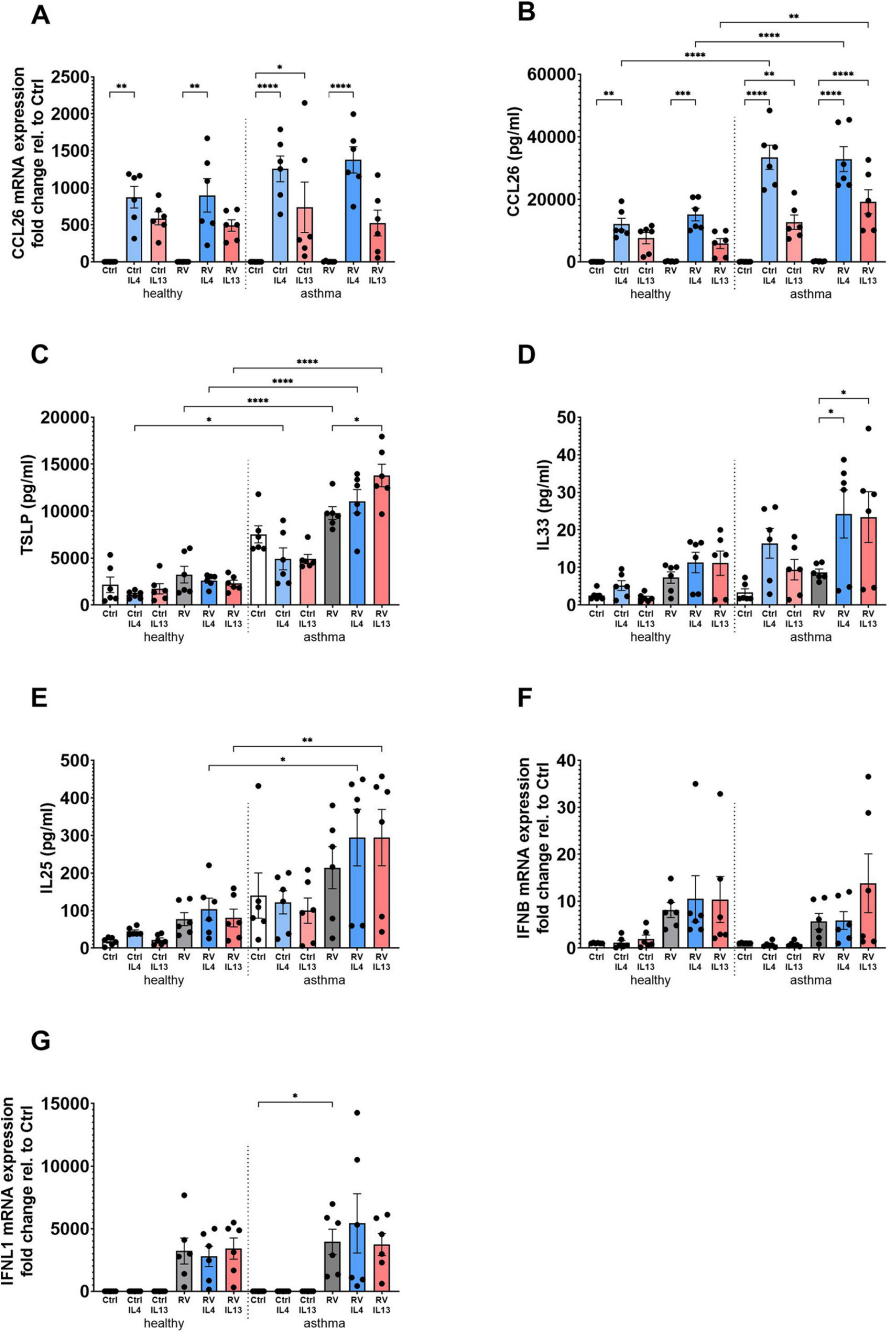
BECs from asthma patients have pronounced inflammatory and antiviral responses following T2 stimulation and RV infection compared to healthy BECs. BECs derived from healthy and asthma donors were stimulated with IL-4 or IL-13 in the presence or absence of RV infection to evaluate mRNA expression **(A)** and protein levels **(B)** of CCL26; protein levels of the epithelial alarmins TSLP **(C)**, IL-33 **(D)**, and IL-25 **(E)**; and antiviral IFNβ **(F)** and IFN*λ*1 **(G)** mRNA expression. Data are presented as mean ± SEM for six healthy and six asthma donors. Statistical methods used are ordinary one-way ANOVA followed by Šidák's multiple-comparisons test with significance indicated as *p < 0.05, **p < 0.01, ****p < 0.0001, or as appropriate.

In BECs derived from healthy and asthma donors, stimulation with IL-4 or IL-13, in the presence or absence of RV, led to a significant upregulation of CCL26 mRNA and protein whereas RV on its own had no effect (Figures 1A,B). Notably, the increase in CCL26 protein levels was significantly higher in BECs from asthma donors compared with healthy donors (Figure 1B). TSLP release in response to RV infection and stimulation with IL-4 was significantly increased in asthmatic BECs as compared with healthy BECs, and the same was observed after stimulation with IL-4 and IL-13 on top of infection with RV (Figure 1C). Similarly, the T2 cytokines in combination with RV-induced IL-33 release in asthmatic BECs but not in healthy BECs (Figure 1D). IL-25 levels were also significantly elevated in asthmatic BECs as compared with healthy BECs following IL-4 or IL-13 stimulation on top of RV infection (Figure 1E). Interferon (IFNβ and IFN*λ*1) gene expression was increased after viral infection in healthy as well as asthmatic BECs, and treatment with IL-4 or IL-13 did not modulate this antiviral response (Figures 1F,G).

### 3.2 IL-4Rα blockade inhibits T2-driven inflammatory responses in bronchial epithelial cells

**Figure 2:**
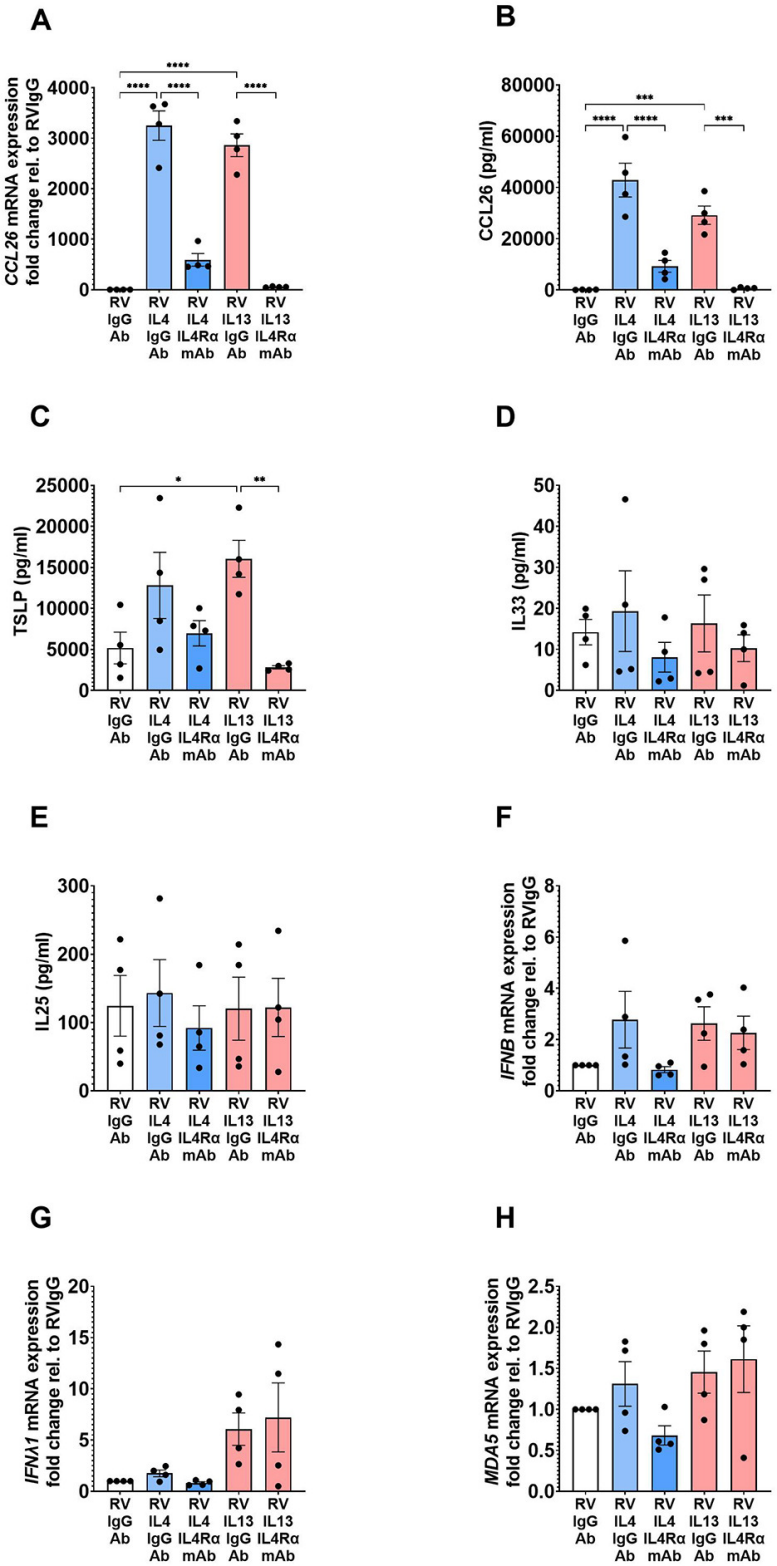
IL-4Rα mAb inhibits CCL26 and TSLP responses following T2 stimulation and RV infection. BECs from asthma donors were treated with IL-4Rα mAb in the presence of IL-4 or IL-13 and RV infection, to evaluate its effects on inflammatory mediator mRNA expression **(A)** and protein **(B)** CCL26; alarmins **(C)** TSLP, **(D)** IL-33, **(E)** IL-25; and antiviral responses **(F)** IFNβ, **(G)** IFN*λ*1, and **(H)** MDA5 gene expression. Data are presented as mean ± SEM for 4 individual donors. Statistical methods used are ordinary one-way ANOVA followed by Šidák's multiple-comparisons test with significance indicated as *p < 0.05, **p < 0.01, ****p < 0.0001, or as appropriate.

We next investigated the effects of an IL-4Rα mAb on the inflammatory (CCL26 and alarmins) and antiviral (interferons and MDA5) responses induced by the T2 cytokines in RV-infected asthmatic BECs. Treatment with IL-4Rα mAb effectively inhibited IL-4- and IL-13-induced CCL26 gene expression (Figure 2A) and protein release (Figure 2B) on background of RV infection. Similarly, IL-13-induced TSLP secretion was significantly reduced by IL-4Rα mAb treatment (Figure 2C), whereas no significant effects of the T2 cytokines, RV infection, or the IL-4Rα mAb on IL-33 and IL-25 were observed (Figures 2D,E). IL-4Rα mAb treatment did not significantly affect the expression of IFNβ, IFN*λ*1, or the cytosolic pattern recognition receptor MDA5 following T2 stimulation and viral infection (Figures 2F–H). Similarly, we could not detect any inhibitory effects by targeting IL-4Rα on the Th1 cytokines; IL-8, IL-6, IL-1β, and TNFα could not detect any inhibitory effects on these Th1 cytokines.

### 3.3 Differential effects of chronic IL-4/IL-13 stimulation on innate immune responses in healthy and asthmatic epithelial cells

**Figure 3:**
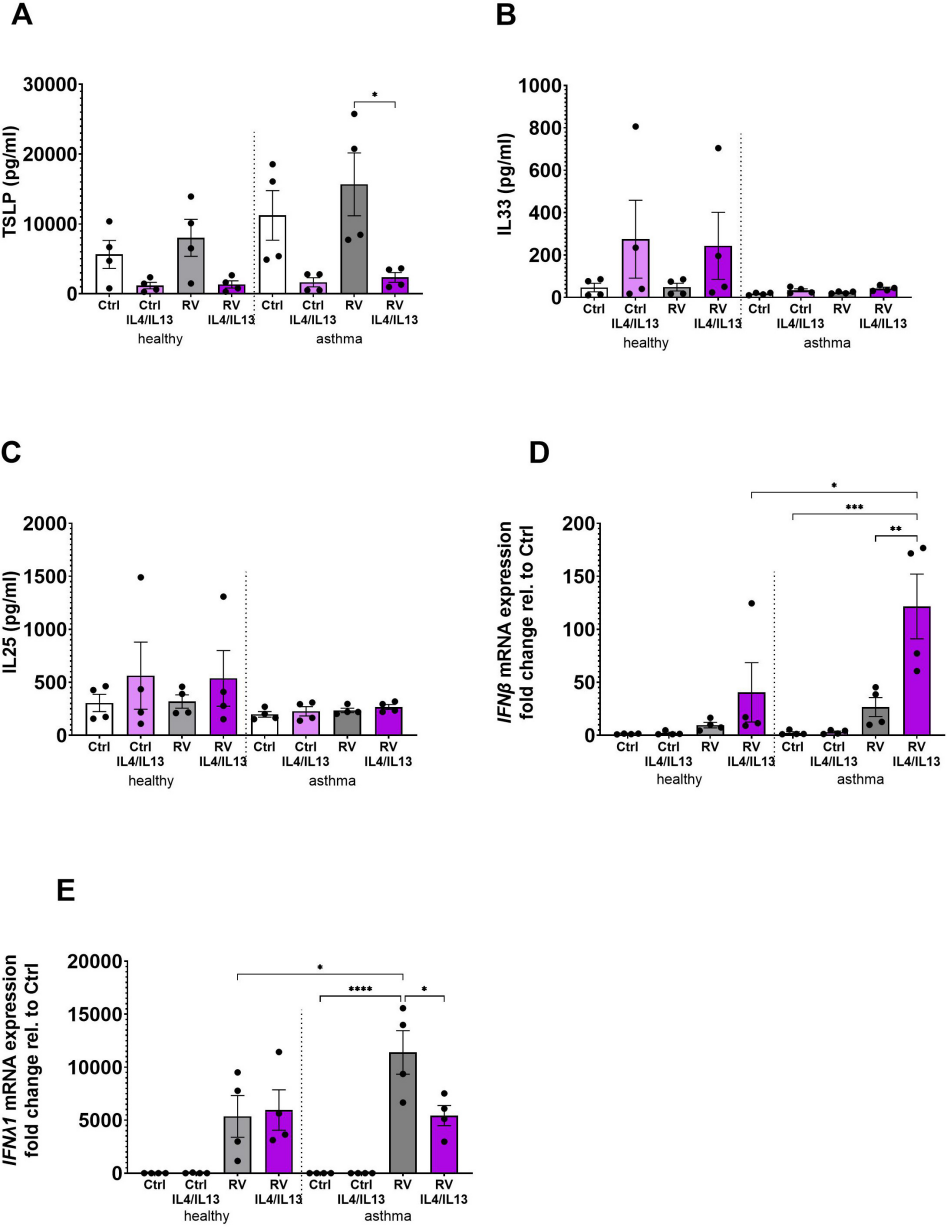
Differential effects of chronic IL-4 and IL-13 stimulation on alarmin and antiviral responses in healthy and asthmatic BECs. BECs from healthy and asthma donors were exposed to IL-4 and IL-13 cytokines for 10 days prior to infection with RV to evaluate its effects on alarmins **(A)** TSLP, **(B)** IL-33, and **(C)** IL-25 and antiviral responses of **(D)** IFNβ and **(E)** IFN*λ*1 gene expression. Data are presented as mean ± SEM for six individual donors. Statistical methods used are ordinary one-way ANOVA followed by Tukey's multiple-comparisons test with significance indicated as *p < 0.05, **p < 0.01, ****p < 0.0001, or as appropriate.

The effects of prolonged IL-4 and IL-13 stimulation on alarmin and antiviral responses were studied in BECs from healthy and asthma donors exposed to a combination of IL-4 and IL-13 for 10 days prior to infection with RV. Contrary to what was observed after short-term stimulation of BECs with T2 cytokines (Figures 1C–E), TSLP secretion was significantly reduced following prolonged T2 cytokine exposure in the presence of virus in asthma BECs (Figure 3A), and the same trend was observed in healthy BECs and non-infected cells (healthy and asthma donors). In contrast, there was a trend toward increased release of IL-33 after chronic T2 cytokine exposure in healthy donor BECs (Figure 3B), whereas the release of IL-25 was not at all modulated by the T2 cytokines or RV under these conditions (Figure 3C). Interestingly, long-term T2 cytokine stimulation followed by viral infection in asthma BECs resulted in a strong upregulation of IFNβ gene expression (Figure 3D), reaching levels significantly higher than in healthy donor BECs. Conversely, the RV-induced IFN*λ*1 gene expression was significantly downregulated after T2 stimulation in asthma BECs (Figure 3E).

## 4 Discussion

Biologics targeting T2-driven immune responses, including dupilumab (anti-IL-4Rα monoclonal antibody), represent a significant advancement in the management of asthma, especially for patients with moderate-to-severe disease where standard treatments may not provide adequate control. Treatment with anti-IL-4Rα antibodies has been shown to significantly reduce the rate of exacerbations and improve lung function in asthmatic patients (17). In the present study, we demonstrated that IL-4Rα mAb effectively modulates the bronchial epithelial immune response to rhinovirus under a type 2 environment (T2 cytokine treatment), reducing CCL26 and TSLP release, without affecting antiviral IFN response. Our study provides valuable insights into the modulation of inflammatory and antiviral responses in human bronchial epithelial cells by the T2 cytokines IL-4 and IL-13 during rhinovirus infection and the effect of IL-4Rα blockade.

Others have previously demonstrated that exposure to T2 cytokines may impair antiviral interferon responses in respiratory epithelial cells following viral challenge (15), suggesting T2 immune responses not only contribute to inflammation of the airways but also increase susceptibility to viral infections. However, despite extensive efforts and varied experimental conditions, we were not able to reproduce this effect of T2 cytokines on the IFN gene expression. Instead, acute and prolonged stimulation of healthy and asthmatic BECs with T2 cytokines tended to increase IFNβ gene expression, indicating that reduced exacerbations in response to treatment with anti-IL-4Rα antibodies are not due to boosting antiviral responses. One possible explanation for the discrepancy between the previously published data and our results could be the use of different sources of bronchial epithelial cells as there is significant variability in the antiviral interferon response between cell donors, which is something we also observe. A potential weakness of our study was that the epithelial cells came from a commercial source with no available data on clinical characteristics and asthma severity which limits the conclusions of this study. The possibility that the asthma patients in our study had a much milder disease may explain the difference between our findings and those of Contoli et al.'s work. Furthermore, in our current research, we focused solely on evaluating changes in IFNβ gene expression and did not include protein measurements. While we initially utilized Meso Scale Diagnostics for our protein assessments, we encountered difficulties in integrating IFNβ into the analysis panel. Moreover, earlier experiments using ELISA indicated that IFNβ levels were undetectable in unstimulated cells. Consequently, we made the informed decision to exclude protein IFNβ from our current study.

Our data suggest that the positive impact of anti-IL-4Rα therapies such as dupilumab on asthma exacerbations in terms of reduced exacerbation frequency and severity is due to inhibition of pro-inflammatory T2 immune (18). This is in line with our observation of significant increases in TSLP, IL-33, and IL-25 protein levels following T2 stimulation and viral infection, specifically in asthma-derived BECs. Bronchial epithelial cells are the primary source of TSLP in the airways. It is well-established that TSLP is overexpressed in T2-driven atopic asthma (19) and that a variety of environmental factors, including mechanical injury, toll-like receptor (TLR) ligands, viruses, and cytokines, can induce its production (20). A particularly interesting finding in our study was the clear differences in TSLP levels between asthma-derived BECs and healthy BECs under T2-driven inflammatory conditions. This overexpression of TSLP in asthma BECs may play a critical role in asthma exacerbations, as TSLP is a key cytokine that amplifies T2 immune responses and promotes airway inflammation (21). Our demonstration that anti-IL-4Rα blockade effectively reduced TSLP secretion by asthma donor BECs acutely stimulated with the T2 cytokines suggests that reduction or normalization of TSLP levels may be an important mechanism, whereby anti-IL-4Rα therapy curbs T2-mediated inflammatory processes, preventing exacerbations in asthma patients.

Stimulation of BECs with the T2 cytokines resulted in robust induction of CCL26, a chemokine known to be activated downstream to stimulation with IL-4 and IL-13 (22). This response was completely blocked by anti-IL-4Rα treatment at both mRNA and protein levels, an observation in line with our previous findings in BECs from chronic obstructive pulmonary disease (COPD) patients (23). Furthermore, T2 cytokine-induced CCL26 secretion was significantly higher in BECs from asthma patients as compared with cells from healthy donors, and since CCL26 is a chemokine critically involved in recruiting eosinophils to the airways, a key feature of airway inflammation in T2-high asthma (24), it emerges as another crucial driver of airway inflammation and hyperresponsiveness targeted by anti-IL-4Rα therapy. The observed increases in eosinophil blood counts observed in dupilumab-treated patients with asthma, chronic rhinosinusitis, and atopic dermatitis (25) may be due to reduced CCL26 release from the airway epithelium disrupting the chemoattractant signal required for blood eosinophils to migrate to the tissue, in turn resulting in increased levels of blood eosinophils. This phenomenon has been observed in a preclinical *in vivo* model, where IL-4Rα blockade prevented lung tissue entry of eosinophils by attenuating chemotaxis and endothelial activation (26, 27). Our findings align with these observations and identify inhibition of CCL26 release from the lung epithelium as one potential mechanism of action behind the therapeutic efficacy of IL-4Rα biologics in asthma. By reducing and normalizing key inflammatory mediators such as CCL26 and TSLP, while preserving essential antiviral defense mechanisms, IL-4Rα blockade offers a balanced approach to managing asthma symptoms and preventing exacerbations.

In Figure 3, we used a more translational approach looking into the combined effects of the T2 cytokines IL-4 and IL-13 as a comparison to stimulation alone. By combining IL-4 and IL-13, we created a more comprehensive model that reflects the complexity of inflammatory processes in lung diseases. Also, this allows us to see how these cytokines can interact synergistically, potentially amplifying certain responses that may not be as pronounced when they are used individually. Chronic exposure to T2 cytokines followed by viral infection revealed a complex interplay between alarmins and antiviral responses in BECs derived from asthma patients and healthy donors. Thus, prolonged treatment with a combination of IL-4 and IL-13 downregulated TSLP secretion and IFN*λ*1 gene expression, while inducing IFNβ expression, especially in BECs from asthma patients. These findings suggest dysregulation of the epithelial immune response during T2-driven inflammation. TSLP is an alarmin with a crucial role in initiating and shaping the immune response to environmental insults including allergens and pathogens (28), and its downregulation under conditions of a chronic T2 immune response may represent a feedback mechanism to suppress or resolve excessive inflammation. Downregulation of TSLP production following chronic T2 cytokine exposure has been observed previously in a study where persistent exposure to IL-13 resulted in decreased TSLP release by asthmatic lung epithelial cells *in vitro* (29). Such T2-mediated downregulation of TSLP release could potentially delay or reduce the severity of virus-induced exacerbations in asthma patients, but on the other hand, the simultaneous decrease in IFN*λ*1, a key antiviral cytokine in epithelial immunity, may impair the airway's ability to mount an effective defense against viral infections. On the contrary, the observed increase in IFNβ suggests a compensatory upregulation of alternative antiviral pathways, to counterbalance impaired IFN*λ*1 signaling. The observed changes in the alarmin and interferon pathways in association with T2 cytokine stimulation and viral infection of lung epithelial cells *in vitro* are highly relevant for asthma, where viral infections in patients with ongoing T2 immune responses are a main driver of exacerbations and disease progression. The complex regulation of alarmin production and antiviral responses in a T2 environment highlights the need for targeted therapies (right patient at the right time) to restore immune balance.

Our work has several limitations. The number of replicates (*N* = 4–6) is low due to due to limited donor availability, making calling of clear effects challenging, especially considering the inherent donor variability with respect to responses to different treatments and stimuli. Still, despite the low number of replicates, the observed effects of treatment with T2 cytokines and anti-IL-4Rα mAb were in most cases clear, with reliable and statistically significant results. We also opted to use the logistically challenging and time-consuming 3D ALI cultures as a model system, which also limited experimental throughput. However, this model much better resembles the lung epithelium *in vivo* features than conventional submerged cultures, increasing the biological relevance of the results obtained. For future studies, we would like to further increase biological relevance by implementing BEC ALI/immune cell co-cultures, enabling cross talk between the epithelial compartment and key immune cells, such as T cells and ILC2 cells.

In conclusion, our findings highlight the interplay between T2-driven inflammation, alarmin production, and antiviral responses in asthma. Anti-IL-4Rα treatment shows promise in mitigating airway inflammation by targeting TSLP, a central mediator of T2 responses. However, the complex shifts in antiviral mediator expression, such as the reduction in IFN*λ*1 and increase in IFNβ, suggest that chronic T2 cytokine exposure may reshape the immune landscape in asthma in a way that requires further exploration.

Future research should address the long-term effects of anti-IL-4Rα therapy on immune tolerance and antiviral defense, as well as its clinical impact on reducing asthma exacerbations. Additionally, investigating the mechanisms underlying the dysregulation of IFN*λ*1 and IFNβ in chronic T2 inflammation may provide new avenues for therapeutic intervention.

## Data Availability

The raw data supporting the conclusions of this article will be made available by the authors, without undue reservation.
