# Supplementary figures and images for: T2 cytokine-driven alarmin and antiviral responses in asthma: insights into immune modulation and the role of IL-4Rα targeting
Source: Front Allergy. 2025 Apr 30;6:1576816. doi: 10.3389/falgy.2025.1576816 (PMC12075527; doi:10.3389/falgy.2025.1576816)

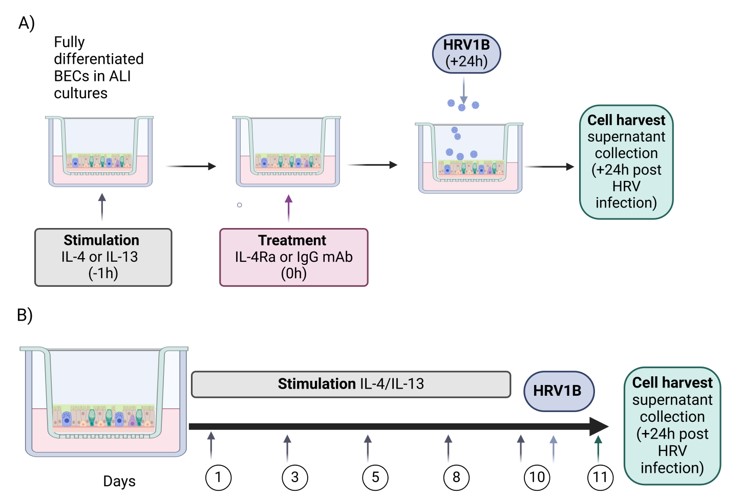

Supplement: Supplementary file 1 [file Image1.jpeg]
